# Supplementary figures and images for: Histological characterization of anther structure in Tetep-cytoplasmic male sterility and fine mapping of restorer-of-fertility gene in rice
Source: PLoS One. 2022 Aug 18;17(8):e0268174. doi: 10.1371/journal.pone.0268174 (PMC9387866; doi:10.1371/journal.pone.0268174)

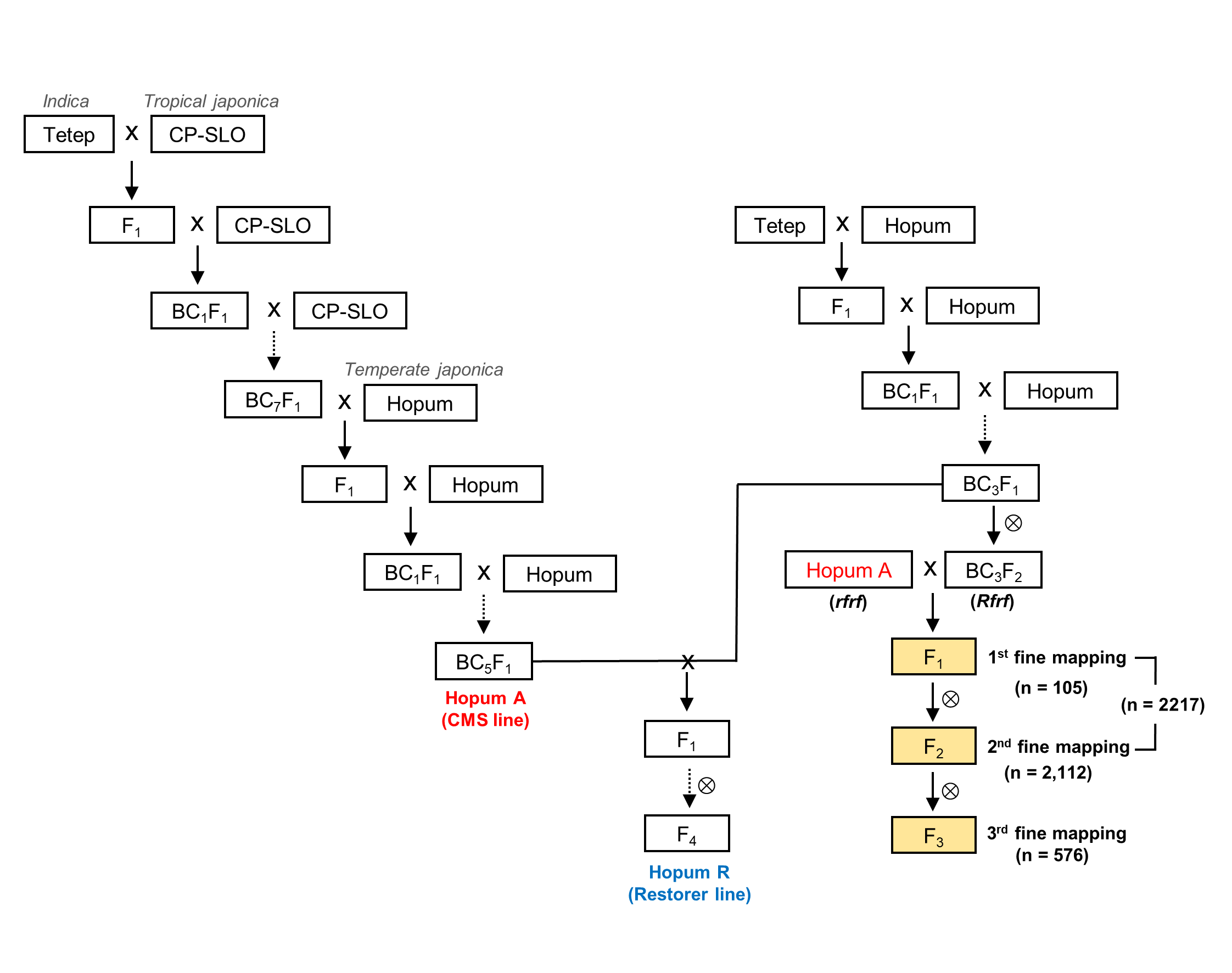

Supplement: S1 Fig — Solid arrow indicates the progeny of a single cross. Dotted arrow indicates successive rounds of crosses. The X mark enclosed within a circle indicates selfing. (TIF) [file pone.0268174.s001.tif]

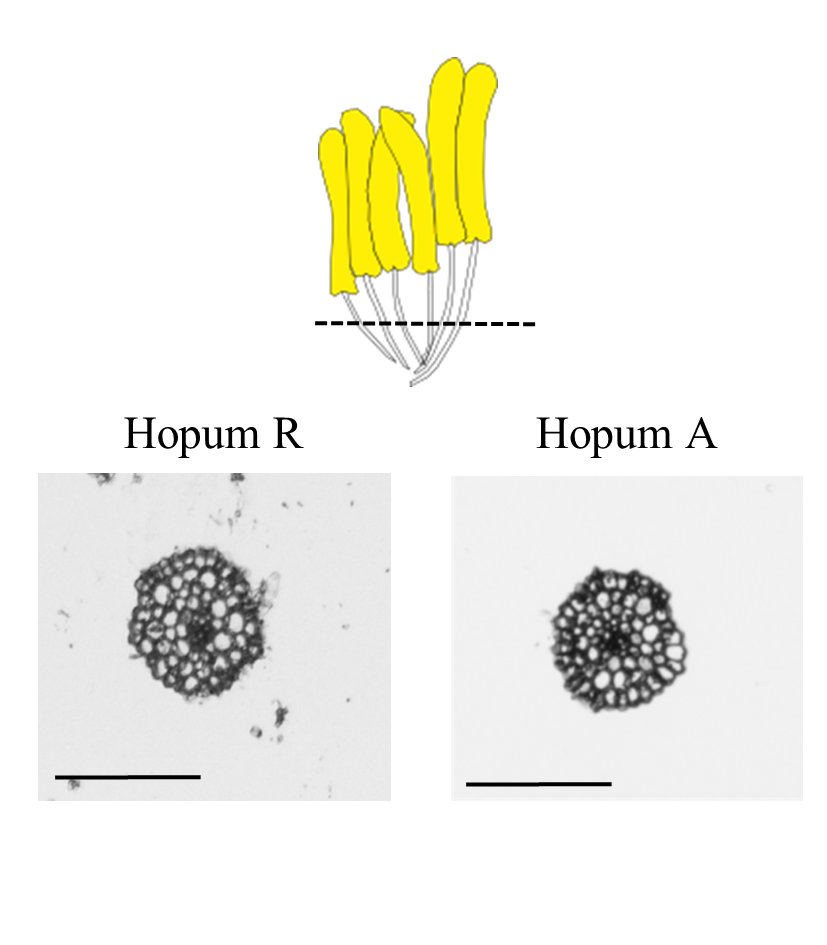

Supplement: S2 Fig — Scale bars = 100 μm. Dotted line represents the position of sectioning. (TIF) [file pone.0268174.s002.tif]

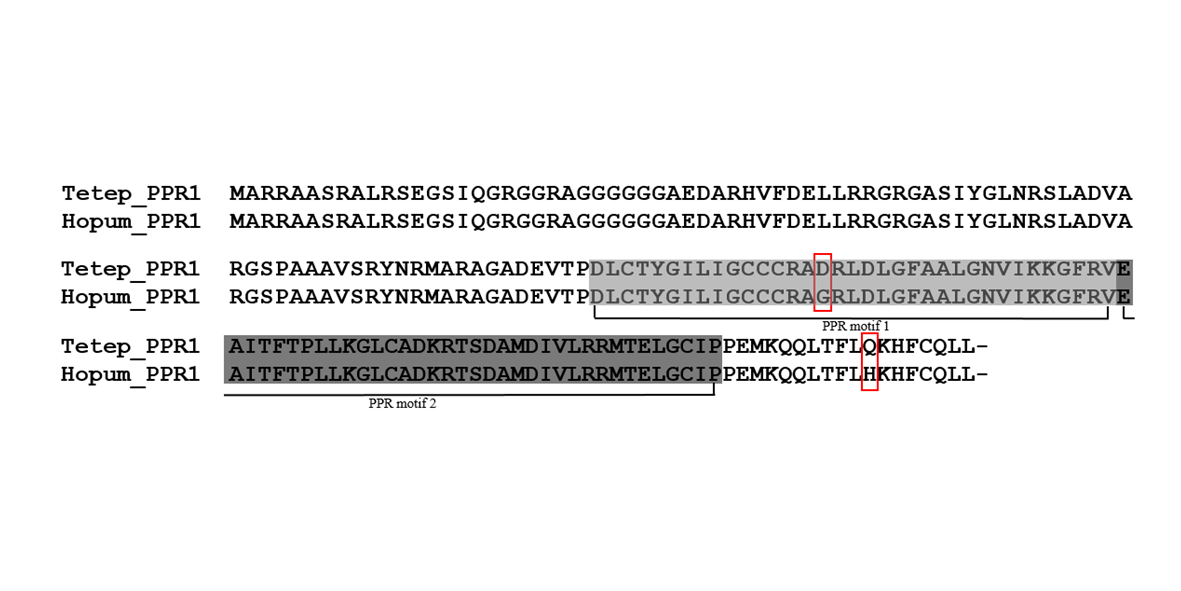

Supplement: S3 Fig — Shaded portion represents the two PPR motifs. (TIF) [file pone.0268174.s003.tif]
