# Supplementary material for: Histological characterization of anther structure in Tetep-cytoplasmic male sterility and fine mapping of restorer-of-fertility gene in rice
Source: PLoS One. 2022 Aug 18;17(8):e0268174. doi: 10.1371/journal.pone.0268174 (PMC9387866; doi:10.1371/journal.pone.0268174)
Supplement: S1 Table — (DOCX) [file pone.0268174.s004.docx]

| Molecular marker | IRGSP position | Hopum R position | Forward (5'-3') | Reverse (5'-3') |
| --- | --- | --- | --- | --- |
| M1801 | 15967823 | 16699386 | GAAACAATTTCCTTTCGGTCA | TCTCAGGGGGTGTGTAATCC |
| M2029 | 18077423 | 18960838 | TGTCCATAGATACGTGCCAATC | AATTGCATCCTTTGCTTGCT |
| M2081 | 18501164 | 19483494 | AGCAAAGCTCCATGCAAGAT | TACTTTGTTTGCCCGTTTGC |
| FM03 | 18667634 | 19650023 | GGGTTTTGCACTTTGATGCT | TTAACAACTACGGCCCGTTT |
| M2099 | 18682221 | 19664568 | ATTCTTTGTGGGGATTGCAG | ATCTGAATGGGAACGACTGG |
| FM04 | 18695964 | 19678241 | AGACAGCGATCTACGCCCTA | GAGAAGCATCAGGGATCAGG |
| FM14 | 18698663 | 19680933 | GCGGATCAGCTACAGTTCT | CTCGACGACGTTCTCATGG |
| FM18 | 18766315 | 19762785 | CAGACACGTCCAAGTCAGTCC | CAAAAGGTCAAGGATGGCATA |
| FM07 | 18791417 | 19787474 | GATAGGGCCGGGTTTAGTTC | CTGCTGTTGCACAATGGTTT |
| M2120 | 18857332 | 19910710 | GGTTGGGGAACTGAAGAGAG | CGTGATGCCCAAAACCTAAT |
| Rf30 | 18875625 | 19930564 | CCACACTTTTTGGTCCATCA | AAGAGGTGGGAGGGGACTTA |
| Rf34 | 18905558 | 19967797 | ATTCCCTGTGGCTCAACAAC | CAGTTTGAAAAACATGCTAATGT |
| Rf35 | 18961881 | 20029124 | TTTGTCCGATAGGCCCTTTA | GAGTCGGTGCAGAATTGGTT |
| Rf32 | 18983704 | 20050452 | TGAGCATGTTGTAGTACATGTGAAA | TGTCAAACCACCCACGTTTA |
| M2147 | 19097968 | 20161012 | TGTCCTGCAACCTTTTGAGTT | TGCCCAAAGAAAAATAGTCACA |
| S10055a | 19596664 | 20617549 | CGTTTGTGACCCCCTTTAGA | TTTTTCAAACGACCCACACA |
| S10078 | 22103505 | 23127499 | GTTCTGATGCGTCCTCCAC | CACACCAGGAGATAACGAGGA |

**S1 Table. List of molecular marker used for fine mapping.**
