# Supplementary material for: Histological characterization of anther structure in Tetep-cytoplasmic male sterility and fine mapping of restorer-of-fertility gene in rice
Source: PLoS One. 2022 Aug 18;17(8):e0268174. doi: 10.1371/journal.pone.0268174 (PMC9387866; doi:10.1371/journal.pone.0268174)
Supplement: S2 Table — (DOCX) [file pone.0268174.s005.docx]

**S2 Table. Agronomic traits of the Tetep-CMS line.**

|  | PH ^ns^ (cm) | TN ^ns^ (No.) | PL ^ns^ (cm) |
| --- | --- | --- | --- |
| Hopum A | 85.4±2.28 | 8.3±0.47 | 17.6±0.41 |
| F_1_ | 90.7±1.79 | 7.0±0.00 | 22.4±0.22 |
| Hopum R | 90.33±0.74 | 7.7±0.47 | 23.6±0.75 |
| Hopum | 86.7±1.59 | 6.0±0.82 | 19.1±0.82 |

PH – Plant height; TN – Tiller number; PL – Panicle length

^ns^ – indicates no significant difference at the 5% confidence level, as verified by a DUNCAN test
