# Supplementary material for: Histological characterization of anther structure in Tetep-cytoplasmic male sterility and fine mapping of restorer-of-fertility gene in rice
Source: PLoS One. 2022 Aug 18;17(8):e0268174. doi: 10.1371/journal.pone.0268174 (PMC9387866; doi:10.1371/journal.pone.0268174)
Supplement: S3 Table — (DOCX) [file pone.0268174.s006.docx]

| Sample name | Total reads | Total read base (bp) | Max length | Average length | N50 |
| --- | --- | --- | --- | --- | --- |
| Hopum R | 3,978,209 | 19,282,005,992 | 139,370 | 4,847 | 9,644 |
| Tetep | 3,350,388 | 14,578,002,439 | 134,366 | 4,351 | 9,155 |
| Hopum | 3,277,590 | 14,548,140,272 | 127,286 | 4,439 | 8,671 |

**S3 Table. Raw data obtained by nanopore long-read sequencing**
