# Supplementary material for: Histological characterization of anther structure in Tetep-cytoplasmic male sterility and fine mapping of restorer-of-fertility gene in rice
Source: PLoS One. 2022 Aug 18;17(8):e0268174. doi: 10.1371/journal.pone.0268174 (PMC9387866; doi:10.1371/journal.pone.0268174)
Supplement: S4 Table — (DOCX) [file pone.0268174.s007.docx]

**S4 Table. Summary of genome assemblies.**

| Statistics | HopumR | Tetep | Hopum |
| --- | --- | --- | --- |
| No. of contigs | 176 | 232 | 237 |
| No. of contigs ≥ 50 kb | 170 | 229 | 233 |
| No. of contigs in chr. 10 | 9 | 16 | 16 |
| Genome assembly length (bp) | 374,938,448 | 384,391,989 | 372,146,397 |
| Chr. 10 length | 24,217,132 | 23,006,168 | 27,320,236 |
| Largest contig size (bp) | 15,686,409 | 13,216,741 | 9,808,537 |
| GC (%) | 43.58 | 43.62 | 43.56 |
| N50 (bp) | 4,732,166 | 4,109,878 | 3,127,046 |
| N75 (bp) | 2,760,600 | 2,045,919 | 1,671,812 |
| L50 | 25 | 30 | 35 |
| L75 | 50 | 64 | 75 |
| Genome coverage (X) | 36.30 | 26.41 | 25.68 |
| BUSCO score (%) | 98.2 | 98.1 | 98.4 |
