# Supplementary material for: Histological characterization of anther structure in Tetep-cytoplasmic male sterility and fine mapping of restorer-of-fertility gene in rice
Source: PLoS One. 2022 Aug 18;17(8):e0268174. doi: 10.1371/journal.pone.0268174 (PMC9387866; doi:10.1371/journal.pone.0268174)
Supplement: S5 Table — (DOCX) [file pone.0268174.s008.docx]

**S5 Table. List of accessions possessing both type 1 genotype and *orf312* sequences.**

| Cultivar ID | Cultivar name | Subpopulation | Location | Accession |
| --- | --- | --- | --- | --- |
| IRIS_313-11734 | SI_CHAO_1 | Indica I | China | ERS468897 |
| IRIS_313-11853 | BU_ZHI_MING | Indica I | China | ERS469006 |
| IRIS_313-11856 | DA_HEI_GU | Indica I | China | ERS469009 |
| IRIS_313-11858 | DA_TIE_ZHAN | Indica I | China | ERS469011 |
| IRIS_313-11950 | MI_GU | Indica I | China | ERS469095 |
| IRIS_313-11953 | SAN_BAO_GU | Indica I | China | ERS469098 |
| IRIS_313-11955 | YE_DA_GU | Indica I | China | ERS469100 |
| CX378 | Huhan_15 | Indica II | China | ERS470664 |
| CX89 | M202 | Indica II | United States | ERS470742 |
| CX79 | Cs94 | Indica II | Vietnam | ERS470732 |
| IRIS_313-10986 | HALDI_JAON | Indica III | Bangladesh | ERS469790 |
| IRIS_313-11113 | KAL_SHULI | Indica III | Bangladesh | ERS469910 |
| IRIS_313-11203 | GOCHIAGARI | Indica III | Bangladesh | ERS469994 |
| IRIS_313-11945 | MALIKULI | Indica III | Bangladesh | ERS469090 |
| IRIS_313-9148 | BADAL_1163 | Indica III | Bangladesh | ERS468030 |
| IRIS_313-11938 | GNASSOUMADOUGOU | Indica III | Burkina Faso | ERS469083 |
| IRIS_313-11939 | KAMPTI | Indica III | Burkina Faso | ERS469084 |
| IRIS_313-7620 | GAMBIAKA | Indica III | Burkina Faso | ERS468642 |
| IRIS_313-10911 | NEANG_NARY | Indica III | Cambodia | ERS469709 |
| IRIS_313-11084 | KABIN | Indica III | Cambodia | ERS469927 |
| IRIS_313-12146 | AM_BEUS | Indica III | Cambodia | ERS469237 |
| IRIS_313-11728 | LUO_SI_ZHAN | Indica III | China | ERS468891 |
| IRIS_313-11773 | AUS_78-125 | Indica III | Gambia | ERS468934 |
| IRIS_313-11783 | MAMA_TABALI | Indica III | Gambia | ERS468939 |
| IRIS_313-10929 | KOLONGI_BAO | Indica III | India | ERS469728 |
| IRIS_313-11271 | ARC_14737 | Indica III | India | ERS470117 |
| CX23 | Rasi | Indica III | Indonesia | ERS470553 |
| IRIS_313-10760 | ATJEH | Indica III | Indonesia | ERS469570 |
| IRIS_313-10768 | GADABUNG(GUNDIL) | Indica III | Indonesia | ERS469576 |
| IRIS_313-10777 | KAMAS | Indica III | Indonesia | ERS469585 |
| IRIS_313-10778 | KENTJANA_MAHANG | Indica III | Indonesia | ERS469586 |
| IRIS_313-10791 | MANGSUR | Indica III | Indonesia | ERS469600 |
| IRIS_313-10792 | MATIANG | Indica III | Indonesia | ERS469601 |
| IRIS_313-10806 | RADEN_DJAWA(GUNDIL) | Indica III | Indonesia | ERS469663 |
| IRIS_313-10807 | RANDA_KUNING | Indica III | Indonesia | ERS469673 |
| IRIS_313-10812 | SERUNEN | Indica III | Indonesia | ERS469614 |
| IRIS_313-10819 | TJERE_ENER | Indica III | Indonesia | ERS469621 |
| IRIS_313-10822 | UNGGAS | Indica III | Indonesia | ERS469625 |
| IRIS_313-11000 | MAIRUL | Indica III | Indonesia | ERS469796 |
| IRIS_313-11010 | PULUT_PUTIH | Indica III | Indonesia | ERS469806 |
| IRIS_313-11179 | SI_OMPAN_LAMA | Indica III | Indonesia | ERS470035 |
| IRIS_313-11320 | MAYAS_PUTIH | Indica III | Indonesia | ERS470112 |
| IRIS_313-11904 | PADI_SIRANDAH_KUNING | Indica III | Indonesia | ERS469047 |
| IRIS_313-12027 | ACEH(SELECTION) | Indica III | Indonesia | ERS469160 |
| IRIS_313-8291 | KEDOT | Indica III | Indonesia | ERS468236 |
| IRIS_313-8608 | SI_HAO_NDURIA | Indica III | Indonesia | ERS468251 |
| IRIS_313-8713 | NELI | Indica III | Indonesia | ERS468257 |
| IRIS_313-8812 | RELLY | Indica III | Indonesia | ERS467976 |
| IRIS_313-9005 | TJERE_SUGI | Indica III | Indonesia | ERS468273 |
| IRIS_313-9117 | BAJAR_KUNING_PAHIT | Indica III | Indonesia | ERS468279 |
| IRIS_313-9188 | TUMBA | Indica III | Indonesia | ERS468281 |
| IRIS_313-9533 | DUD_KUNING | Indica III | Indonesia | ERS468080 |
| IRIS_313-9989 | MELEKE | Indica III | Ivory Coast | ERS468138 |
| IRIS_313-11810 | PULURE | Indica III | Kenya | ERS468959 |
| IRIS_313-11812 | ASFALA | Indica III | Kenya | ERS468962 |
| IRIS_313-11813 | ZERO | Indica III | Kenya | ERS468963 |
| IRIS_313-11814 | KIKUBA | Indica III | Kenya | ERS468964 |
| IRIS_313-11815 | MOSHI | Indica III | Kenya | ERS468965 |
| IRIS_313-11108 | NONE | Indica III | Liberia | ERS469904 |
| IRIS_313-11160 | NONE | Indica III | Liberia | ERS469962 |
| CX141 | Padi_Siam_Kuning | Indica III | Malaysia | ERS470505 |
| CX71 | Innmayebaw | Indica III | Malaysia | ERS470724 |
| IRIS_313-8591 | SIAM_ER_32 | Indica III | Malaysia | ERS468249 |
| IRIS_313-11128 | GOKAUNG | Indica III | Myanmar | ERS469926 |
| IRIS_313-11135 | MAUNG_NYO | Indica III | Myanmar | ERS469934 |
| IRIS_313-11138 | NGASEIN_THEEDAT(C_30) | Indica III | Myanmar | ERS469937 |
| IRIS_313-11150 | ZEINGYI | Indica III | Myanmar | ERS469951 |
| IRIS_313-11406 | A_28-6 | Indica III | Myanmar | ERS470198 |
| IRIS_313-12286 | EKAYIN_SAW | Indica III | Myanmar | ERS469303 |
| IRIS_313-8697 | YEBAWYIN | Indica III | Myanmar | ERS467965 |
| IRIS_313-11565 | KUSUM_KATIKI | Indica III | Nepal | ERS468760 |
| IRIS_313-10825 | KEMA_5 | Indica III | None | ERS469628 |
| IRIS_313-9825 | MOCHICA | Indica III | Peru | ERS468119 |
| IRIS_313-10724 | MOUSOIR | Indica III | Senegal | ERS469530 |
| IRIS_313-10727 | KOUPENEDOU_KOUBOURY | Indica III | Senegal | ERS469534 |
| IRIS_313-11555 | PA_WOON | Indica III | Sierra Leone | ERS468752 |
| IRIS_313-11779 | KATUMANI | Indica III | Tanzania | ERS468936 |
| IRIS_313-8909 | FAYA_MOSHI | Indica III | Tanzania | ERS467987 |
| IRIS_313-11534 | NAHNG_KHAO_BOW | Indica III | Thailand | ERS468732 |
| IRIS_313-11684 | OB_CHUEY | Indica III | Thailand | ERS468859 |
| IRIS_313-11836 | KHAO_KO_RAOH | Indica III | Thailand | ERS468988 |
| IRIS_313-8474 | PAH_WEAN | Indica III | Thailand | ERS467946 |
| IRIS_313-8586 | PLI_KHAO | Indica III | Thailand | ERS467953 |
| IRIS_313-8702 | KHAO_PRAHJIN | Indica III | Thailand | ERS468255 |
| IRIS_313-8785 | LEUANG_AWN | Indica III | Thailand | ERS468265 |
| IRIS_313-9281 | KHAO_KAI | Indica III | Thailand | ERS468050 |
| IRIS_313-10516 | NONE | Indica III | United States | ERS469390 |
| IRIS_313-11254 | TIEN_SOM | Indica III | Vietnam | ERS470049 |
| IRIS_313-10966 | HURANG_ARISO_LUTA | Indica admixture | Brazil | ERS469768 |
| B075 | Jinbaoyin_ | Indica admixture | China | ERS470289 |
| B076 | Minbeiwanxian | Indica admixture | China | ERS470290 |
| B085 | Xugunuo | Indica admixture | China | ERS470297 |
| B129 | Hongainuo | Indica admixture | China | ERS470339 |
| B133 | Yanshuichi | Indica admixture | China | ERS470343 |
| B207 | Aihechi | Indica admixture | China | ERS470410 |
| B227 | Menjiagao_1 | Indica admixture | China | ERS470427 |
| B265 | Wukezhan_ | Indica admixture | China | ERS470459 |
| CX19 | Zhong_413 | Indica admixture | China | ERS470532 |
| CX386 | NPT-114 | Indica admixture | China | ERS470672 |
| IRIS_313-10221 | BA_BAI_GU | Indica admixture | China | ERS468176 |
| IRIS_313-10477 | RUSTY_LATE | Indica admixture | China | ERS469372 |
| IRIS_313-10614 | HAM_MOON | Indica admixture | China | ERS469460 |
| IRIS_313-11573 | GAO_LIANG_ZAO | Indica admixture | China | ERS468767 |
| IRIS_313-11577 | LANG_QIAN_CHE | Indica admixture | China | ERS468771 |
| IRIS_313-11580 | TAI_ZHOU_XIAN | Indica admixture | China | ERS468774 |
| IRIS_313-11693 | CHING-CH'UNG | Indica admixture | China | ERS468868 |
| IRIS_313-11694 | CHING-LIU | Indica admixture | China | ERS468869 |
| IRIS_313-11729 | MEI_LIU_ZAO_5 | Indica admixture | China | ERS468892 |
| IRIS_313-11735 | YI_LI_ZHONG | Indica admixture | China | ERS468898 |
| IRIS_313-11744 | AI_JIAO_AO_FAN_ZI | Indica admixture | China | ERS468908 |
| IRIS_313-11748 | GAO_JIAO_YING_GAN_ZHAN | Indica admixture | China | ERS468912 |
| IRIS_313-11910 | YU_HE_HONG | Indica admixture | China | ERS469053 |
| IRIS_313-11947 | BIE_LEI | Indica admixture | China | ERS469092 |
| IRIS_313-11967 | TANG_DU_GU | Indica admixture | China | ERS469113 |
| IRIS_313-12007 | JIA_GEN | Indica admixture | China | ERS469145 |
| IRIS_313-12011 | YU_TOU_ZHONG | Indica admixture | China | ERS469149 |
| IRIS_313-12057 | AI_JIAO_ZI | Indica admixture | China | ERS469189 |
| IRIS_313-12232 | TE_SAN_AI_2 | Indica admixture | China | ERS469270 |
| IRIS_313-12275 | MAGU | Indica admixture | China | ERS469293 |
| IRIS_313-8405 | JIN_JUN_DAO | Indica admixture | China | ERS467836 |
| IRIS_313-8743 | NIAO_YAO | Indica admixture | China | ERS467838 |
| IRIS_313-9184 | XI_GAN_JING_REN | Indica admixture | China | ERS468033 |
| IRIS_313-9555 | CHUA-DAU | Indica admixture | China | ERS467792 |
| IRIS_313-11134 | MANSAT-3 | Indica admixture | Myanmar | ERS469933 |
| CX42 | IR68552-55-3-2 | Indica admixture | Philippines | ERS470690 |
| CX64 | IRAT_352 | Indica admixture | Philippines | ERS470717 |
| B184 | CHANH_148 | Indica admixture | Vietnam | ERS470389 |
| IRIS_313-10751 | LUA_CHUM | Indica admixture | Vietnam | ERS469560 |
| IRIS_313-11383 | CHIEM_NGAN | Indica admixture | Vietnam | ERS470175 |
| IRIS_313-11384 | CHIEM_TONG_NHAT_1 | Indica admixture | Vietnam | ERS470176 |
| IRIS_313-11893 | KHAU_DANH | Indica admixture | Vietnam | ERS469120 |
| IRIS_313-8341 | BAT_DO | Indica admixture | Vietnam | ERS467930 |
| IRIS_313-8409 | SOM_NGHE_AN | Indica admixture | Vietnam | ERS467793 |
| IRIS_313-9429 | RTS16 | Indica admixture | Vietnam | ERS468290 |
