# Supplementary material for: Histological characterization of anther structure in Tetep-cytoplasmic male sterility and fine mapping of restorer-of-fertility gene in rice
Source: PLoS One. 2022 Aug 18;17(8):e0268174. doi: 10.1371/journal.pone.0268174 (PMC9387866; doi:10.1371/journal.pone.0268174)
Supplement: Attachment — Submitted filename: PlosOne_D2202455.pdf [file pone.0268174.s009.pdf]

The manuscript “Histological characterization of anther structure in Tetep-cytoplasmic male sterility and fine mapping of restorer-of-fertility gene in rice” by Lee et al. covers on traits required to produce hybrids in self-pollinated species (in this case rice) using a cytoplasmic male sterility system. The CMS system characterized in this study is unique because the pollen is functional, but the anthers are unable to dehisce therefore leaving the plant sterile. Further research on this system is intriguing as it offers a unique opportunity for hybrid breeders if a way could be found to chemically dehisce the anthers to allow self-pollination therefore eliminating the need for maintainer line development. This study also evaluated the restorer gene(s) for this CMS system by creating a F3 mapping population for fine mapping and utilizing long read sequencing to characterize the region of interest. Very interestingly, the Rf gene appears to operate due to allelic differences between subspecies.

Overall, I very much enjoyed this paper. The methods are well designed, and modern genomics tools are combined with traditional biparental mapping to elucidate Rf genes. I recommend some minor revisions listed below. Congratulations on your work. The unique results could be very useful to hybrid crop breeders in multiple small grains species.

Suggested revisions

Introduction-

Line 61 Briefly explaining WA352c will help readers understand the process. E.g. the type of protein and where it accumulates in the anthers.

Line 81-87 If the genomic sequencing referred to in these lines was completed as part of the previously published research, you will need to differentiate from the sequencing presented here. If it is the same dataset, it should be cited as such for clarity.

M&M-

Line 99 The S1 Fig seems to indicate that the cross was between a BC3F2. Which is correct the figure or text?

Line 117 Was the pollen tested to confirm viability?

Is line 133-134 referring to extracting the mapping population or the parents or both? It is a little confusing.

Line 136- Please include the number of markers designed in this line. Based on my reading of the current text, I thought it was only one, which obviously wouldn’t work for fine mapping

Results-

Line 211 and throughout the paper- The terms Hopum A and Tetep-CMS line are used interchangeably throughout the manuscript, and it becomes confusing for the reader. Figure 1 is a really beautiful figure though! Congratulations on the very well-done photos.

Line 223 It wasn’t clear that you had switched back to the Hopum R line here, so I recommend making sure to clearly specify which line you are talking about throughout this paragraph.

Line 315 should be “derived”?

You have quite a few acronyms in this paper and it can get confusing. Please consider writing out names rather than using acronyms only a couple of times e.g. RAP-DB, ROS

Line 409 convincible enough? Not sure what you are saying.
