# Supplementary material for: Histological characterization of anther structure in Tetep-cytoplasmic male sterility and fine mapping of restorer-of-fertility gene in rice
Source: PLoS One. 2022 Aug 18;17(8):e0268174. doi: 10.1371/journal.pone.0268174 (PMC9387866; doi:10.1371/journal.pone.0268174)
Supplement: Attachment — Submitted filename: Review.docx [file pone.0268174.s010.docx]

This study investigated the Histological characterization of anther structure in Tetep-cytoplasmic male sterility and fine mapping of restorer-of-fertility gene in rice. I think that the reported results are interesting. However, some more data are required.

1. In this paper, a lot of work has been done. However, please add pollen germination experiments
2. There are some typos and English style problems throughout the text that should be carefully reviewed.
