# Supplementary material for: Histological characterization of anther structure in Tetep-cytoplasmic male sterility and fine mapping of restorer-of-fertility gene in rice
Source: PLoS One. 2022 Aug 18;17(8):e0268174. doi: 10.1371/journal.pone.0268174 (PMC9387866; doi:10.1371/journal.pone.0268174)
Supplement: Attachment — Submitted filename: Response to Reviewers.docx [file pone.0268174.s011.docx]

Response to reviewers’ comments

Reviewer #1

The manuscript “Histological characterization of anther structure in Tetep-CMS and fine mapping of Rf gene in rice” is going to report the characterizations of Tetep-CMS in rice and their restore gene Rf. It is an interesting paper for readers. The manuscript is prepared well.

**Q.** The only concern of manuscript is the conclusion for candidate. There is no any experiment result to support which gene in the mapping interval is the candidate for Rf gene. Even in the discussion the authors didn’t list a number of literatures to convince us which gene must be the possible candidate for Rf. In this situation, I suggested that the author should to modify the conclusion: “suggest ORF6 annotated as pentatricopeptide repeat motif containing gene 1 (PPR1), as the candidate gene responsible for fertility restoration”.

**Response:** Thank you for your valuable comment. We tried to emphasize that majority of discovered *Rf* genes are PPR proteins in the introduction and added supportive information that the PPR proteins suppress the detrimental effects on several CMS-related proteins in our discussion. The main reason we concluded *PPR1* as the candidate gene is because it is the only PPR gene present within the candidate region based on our fine mapping results. However, we also agree with your concern, therefore, we have revised our conclusion as “suggest ORF6 annotated as pentatricopeptide repeat motif containing gene 1 (PPR1), as a possible candidate gene responsible for fertility restoration”.

**Q.** In addition, the Rf gene here reported should be renamed as Rf? (Rf7 or something else, depending how many Rf genes have cloned so far in rice).

**Response:** Thank you for your comment. We have already reported as *Rf*-Tetep on our previous study of “Jin Z, Seo J, Kim B, Lee SY, Koh HJ. Identification of a Candidate Gene for the Novel Cytoplasmic Male Sterility Derived from Inter-Subspecific Crosses in Rice (Oryza sativa L.). Genes (Basel). 2021;12(4),” and cited as well in “Takatsuka, A., Kazama, T., Arimura, S.-i. and Toriyama, K. (2022), TALEN-mediated depletion of the mitochondrial gene *orf312* proves that it is a Tadukan-type cytoplasmic male sterility-causative gene in rice. Plant J.” To avoid confusion, we would like to maintain the gene nomenclature but considering its renaming after positional cloning and complementary experiments. Please consider this.

Reviewer # 2

Please see attached comments for all of my revision recommendations. This is an excellent paper and very interesting research. All criteria for publication after minor revision are met. Thank you for publishing!

The manuscript “Histological characterization of anther structure in Tetep-cytoplasmic male sterility and fine mapping of restorer-of-fertility gene in rice” by Lee et al. covers on traits required to produce hybrids in self-pollinated species (in this case rice) using a cytoplasmic male sterility system. The CMS system characterized in this study is unique because the pollen is functional, but the anthers are unable to dehisce therefore leaving the plant sterile. Further research on this system is intriguing as it offers a unique opportunity for hybrid breeders if a way could be found to chemically dehisce the anthers to allow self-pollination therefore eliminating the need for maintainer line development. This study also evaluated the restorer gene(s) for this CMS system by creating a F3 mapping population for fine mapping and utilizing long read sequencing to characterize the region of interest. Very interestingly, the Rf gene appears to operate due to allelic differences between subspecies.

Overall, I very much enjoyed this paper. The methods are well designed, and modern genomics tools are combined with traditional biparental mapping to elucidate Rf genes. I recommend some minor revisions listed below. Congratulations on your work. The unique results could be very useful to hybrid crop breeders in multiple small grains species.

**Response:** Thank you for your detailed comments. Remarks you have given us are truly inspirational and insightful which will definitely help us to develop our further studies. Your kind compliments have encouraged us to improve in the future.

Suggested revisions

Introduction

**Q.** Line 61 Briefly explaining WA352c will help readers understand the process. E.g. the type of protein and where it accumulates in the anthers.

**Response**: Thank you for your suggestion. We have added description of WA352c in line 61-62.

**Q.** Line 81-87: If the genomic sequencing referred to in these lines was completed as part of the previously published research, you will need to differentiate from the sequencing presented here. If it is the same dataset, it should be cited as such for clarity.

**Response**: Thank you for your detailed advice. We have moved the citation at the end of the paragraph for the clarification.

M&M

**Q.** Line 99 The S1 Fig seems to indicate that the cross was between a BC3F2. Which is correct the figure or text?

**Response**: We appreciate for finding our typo. The figure is correct. We have corrected our error on line 99.

**Q.** Line 117: Was the pollen tested to confirm viability?

**Response**: We have tested to confirm the pollen viability. Kindly refer to our previous report of “Jin Z, Seo J, Kim B, Lee SY, Koh H-J. Identification of a Candidate Gene for the Novel Cytoplasmic Male Sterility Derived from Inter-Subspecific Crosses in Rice (*Oryza sativa* L.). Genes. 2021; 12(4):590. https://doi.org/10.3390/genes12040590” Figure1 i and j.

**Figure 1.** Phenotypes of plants. From left to right: Hopum, Hopum A, F_1_ (Hopum A/Hopum R), Hopum R, Hopum, and Hopum A. (**a**) Plants before harvest. The scale bar = 30 cm; (**b**) spikelet and anthers after spikelet closure; (**c**–**f**) pollen tubes can be observed in the ovule, the black arrow point to pollen tubes; (**g**,**h**) pollen staining with I_2_-KI; (**i**,**j**) in vitro pollen germination with germination medium; (**k**,**l**) pollen tube growth on the stigma, the black arrows point to pollen tubes in (**k**). F_1_: derived from Hopum A/Hopum R.

**Q.** Is line 133-134 referring to extracting the mapping population or the parents or both? It is a little confusing.

**Response**: Thank you for your comment. Line 133-134 refers to both mapping population and the parents which are used for fine mapping.

**Q.** Line 136- Please include the number of markers designed in this line. Based on my reading of the current text, I thought it was only one, which obviously wouldn’t work for fine mapping

**Response**: Thank you for your advice. We included the number of markers designed on line 136 (S1 Table).

Results

**Q.** Line 211 and throughout the paper- The terms Hopum A and Tetep-CMS line are used interchangeably throughout the manuscript, and it becomes confusing for the reader. Figure 1 is a really beautiful figure though! Congratulations on the very well-done photos.

**Response:** Thank you very much for your compliment. The term Hopum A was used when the result is derived from the plant data (such as phenotypic characteristics and HMW DNA used for long-read sequencing). While, ‘Tetep-CMS line’ was used to when it needs to be distinguished among other restorer and maintainer lines.

**Q.** Line 223 It wasn’t clear that you had switched back to the Hopum R line here, so I recommend making sure to clearly specify which line you are talking about throughout this paragraph.

**Response:** Thank you for your comment. Line 223 starts with Hopum R because Fig2A starts by describing the histological characteristics of Hopum R during early stage of anthesis.

**Q.** Line 315 should be “derived”? You have quite a few acronyms in this paper and it can get confusing. Please consider writing out names rather than using acronyms only a couple of times e.g. RAP-DB, ROS

**Response**: Thank you for pointing out our error. We remove ‘-un’ on line 315.

**Q.** Line 409 convincible enough? Not sure what you are saying.

**Response**: Thank you for your comment. We have revised line as ‘not equitable enough’ for clearer explanation.

Reviewer # 3

The group of Hee-Jong Koh employed the Tetep-CMS/ Rf lines to identify a pentatricopeptide repeat motif containing gene 1 (PPR1) in Rice. Nanopore long-read sequencing-based genome assembly to obtain genomic information and conducted fine mapping using a series of segregating populations. Genetics and phenotypic analysis of CMS plants and its downstream targets suggested that tissue-specific abnormalities in anthers are responsible for indehiscence-based sterility, and propose that the functional Rf gene is derived from allelic variation between inter-subspecies in rice. The experiments and analyses are generally conducted in a solid way, and the article is also clearly written. I believe it can meet the publication requirements after minor revision. There are three minor points I would like to address here:

**Response**: Thank you for your detailed remarks.

**Q.** 1. The most important thing I am concerned with is that the Nanopore long-read sequencing of genomes were generated for identified the causal gene. This is a important because, for lots of natural mutation in plant, NGS technology such as Illumina Hiseq is not power enough to identified large InDels that lead to disrupt of the causal gene. So this should be emphasized in this paper. I think besides the discovery of an unannotated Tetep-specific segment with features of a retrotransposon within the region. The author can analyze large InDels, SVs between the three sequenced materials, and give an analysis and discussion that why other InDels and SVs is not associated with the CMS phenotype.

**Response**: We appreciate your precise review. Our initial approach when identifying candidate gene(s) was to analyze large InDels and structural variations between the assembled genome using ONT long-read sequencing data. However, based on our fine mapping results, large InDels were not included within the candidate region and there was no significant structural variation within the candidate region aside from the Tetep-specific segment which features retrotransposon. Analysis of large InDels outside the candidate region where PPR genes are located can be conducted in an independent study. Kindly consider this.

**Q.** 2. Table 1 can move to supplementary data, because it is just basic statistics for sequencing. I suggested the author show the genomic variations and their effect in the main text using table or figure.

**Response:** Thank you for your comment. We moved Table 1 to supplementary table (S4 Table).

3. The stages of anther development have clear definition and standard names in rice. Please refer to the paper from Zhang Dabing’s lab such as “Dasheng Zhang, Wanqi Liang, Changsong Yin, Jie Zong, Fangwei Gu, Dabing Zhang, OsC6, Encoding a Lipid Transfer Protein, Is Required for Postmeiotic Anther Development In Rice, Plant Physiology, Volume 154, Issue 1, September 2010, Pages 149–162, https://doi.org/10.1104/pp.110.158865”. Revised Figure 2 and related content.

**Response:** Thank you for your detailed review. We referred the suggested paper from Zhang Dabing’s lab and revised the figure and related content.

One defect of this research is that they did not verify the causal gene using genetic engineering such as CRISPR/Cas9 genome-editing. If they can add this result, it would be better.

**Response**: Thank you for your comment. We are currently working on transgenic experiments for gene validation.

Reviewer # 4

This study investigated the Histological characterization of anther structure in Tetep-cytoplasmic male sterility and fine mapping of restorer-of-fertility gene in rice. I think that the reported results are interesting. However, some more data are required.

**Response**: Thank you for your kind remarks.

**Q.** In this paper, a lot of work has been done. However, please add pollen germination experiments

**Response**: Thank you for your comment. We have already reported in vitro pollen germination test from our previous study. Kindly refer to figure1 i and j from “Jin Z, Seo J, Kim B, Lee SY, Koh H-J. Identification of a Candidate Gene for the Novel Cytoplasmic Male Sterility Derived from Inter-Subspecific Crosses in Rice (*Oryza sativa* L.). Genes. 2021; 12(4):590. https://doi.org/10.3390/genes12040590”

**Figure 1.** Phenotypes of plants. From left to right: Hopum, Hopum A, F_1_ (Hopum A/Hopum R), Hopum R, Hopum, and Hopum A. (**a**) Plants before harvest. The scale bar = 30 cm; (**b**) spikelet and anthers after spikelet closure; (**c**–**f**) pollen tubes can be observed in the ovule, the black arrow point to pollen tubes; (**g**,**h**) pollen staining with I_2_-KI; (**i**,**j**) in vitro pollen germination with germination medium; (**k**,**l**) pollen tube growth on the stigma, the black arrows point to pollen tubes in (**k**). F_1_: derived from Hopum A/Hopum R.

2. There are some typos and English style problems throughout the text that should be carefully reviewed.

**Response**: Thank you for concern. We requested professional English editing services and further reviewed and corrected some of the typos.
